# A Rare Case of Congenital Absence of Flexor Digitorum Profundus in the Index Finger

**DOI:** 10.7759/cureus.99007

**Published:** 2025-12-11

**Authors:** Shilpi Karmakar, Deepti Katrolia, Dipika Choudhary

**Affiliations:** 1 Burns and Plastic Surgery, All India Institute of Medical Sciences, Jodhpur, IND; 2 Plastic Surgery, All India Institute of Medical Sciences, Jodhpur, IND

**Keywords:** cadaver, contracture, flexor digitorum profundus, flexor tendon rupture, outpatients, prevalence, tendon transfer, young adult

## Abstract

Congenital anomalies of the flexor tendons in the human hand are rare, and those of the flexor digitorum profundus tendon (FDPt) are even rarer. Our search of the literature unearthed only eleven studies, with only one other report of absent FDPt in the index finger. This study presents a case of a 19-year-old male with congenital absence of FDPt in the index finger, discovered incidentally while seeking treatment for post-burn flexion contractures in the ring and little finger. The girth of the distal phalange was reduced compared to that of the opposite finger, and the distal digital crease was absent. Magnetic resonance imaging confirmed the diagnosis, with no compensatory tendon anomalies. The patient underwent release of post-burn flexion contractures with multiple Z-plasties and refused treatment for the index finger. These anatomic variants may cause misinterpretation during the evaluation of a patient with injuries. This case focuses on the need for thorough preoperative assessment.

## Introduction

Congenital anomalies of the flexor tendons in the human hand are uncommon; anomalies affecting the flexor digitorum profundus tendon (FDPt) are even rarer. The FDP muscle originates from the ulna and interosseous membrane and inserts on the distal phalanges of the index, middle, ring, and little fingers. Its action is to flex the fingers at the distal interphalangeal joint and also at the proximal interphalangeal and metacarpophalangeal joints. Anomalies of FDPt are underreported due to their asymptomatic nature [1]. The principal flexors of the proximal interphalangeal and metacarpophalangeal joints are flexor digitorum superficialis (FDSt) and lumbricals, respectively. Absence of one of these tendons usually does not interfere with the daily activities of the patient. Being congenital, patients usually compensate for the lack of one tendon by overusing another tendon. However, knowledge of these anomalies is essential for the identification of the condition in patients with concomitant injuries. The little finger is most commonly afflicted, with lower incidences reported in other digits [2]. We report a rare case of the absence of FDPt on the index finger.

## Case presentation

**Figure 1:**
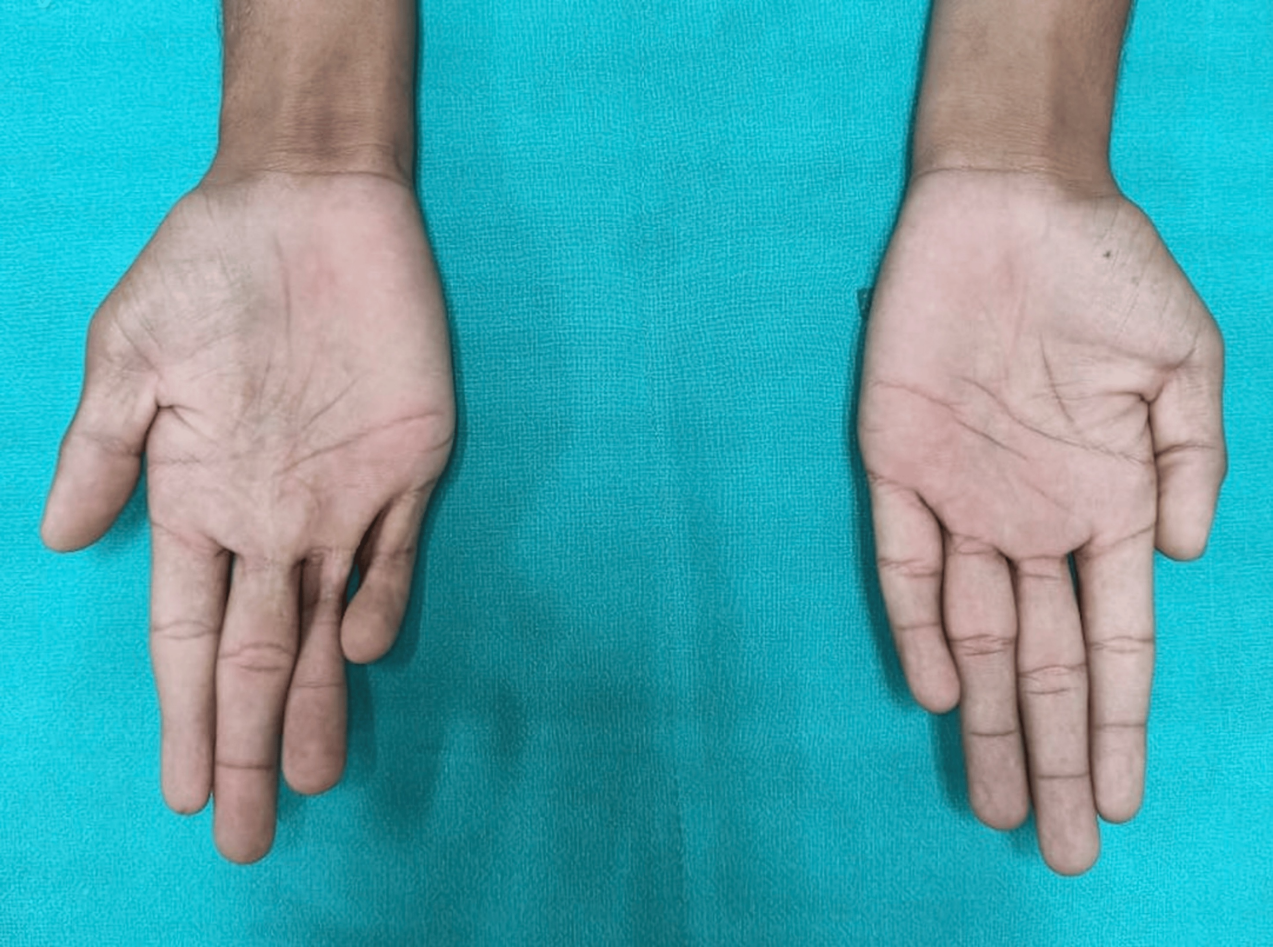
Volar picture of both hands, showing flexion contracture of right ring and little finger, difference in girth of distal index fingers and absence of distal crease in right index finger

A 19-year-old male, left-dominant, presented to the outpatient department with complaints of inability to completely extend his right ring and little finger, after sustaining burns to these fingers in childhood (Figure 1).

**Figure 2:**
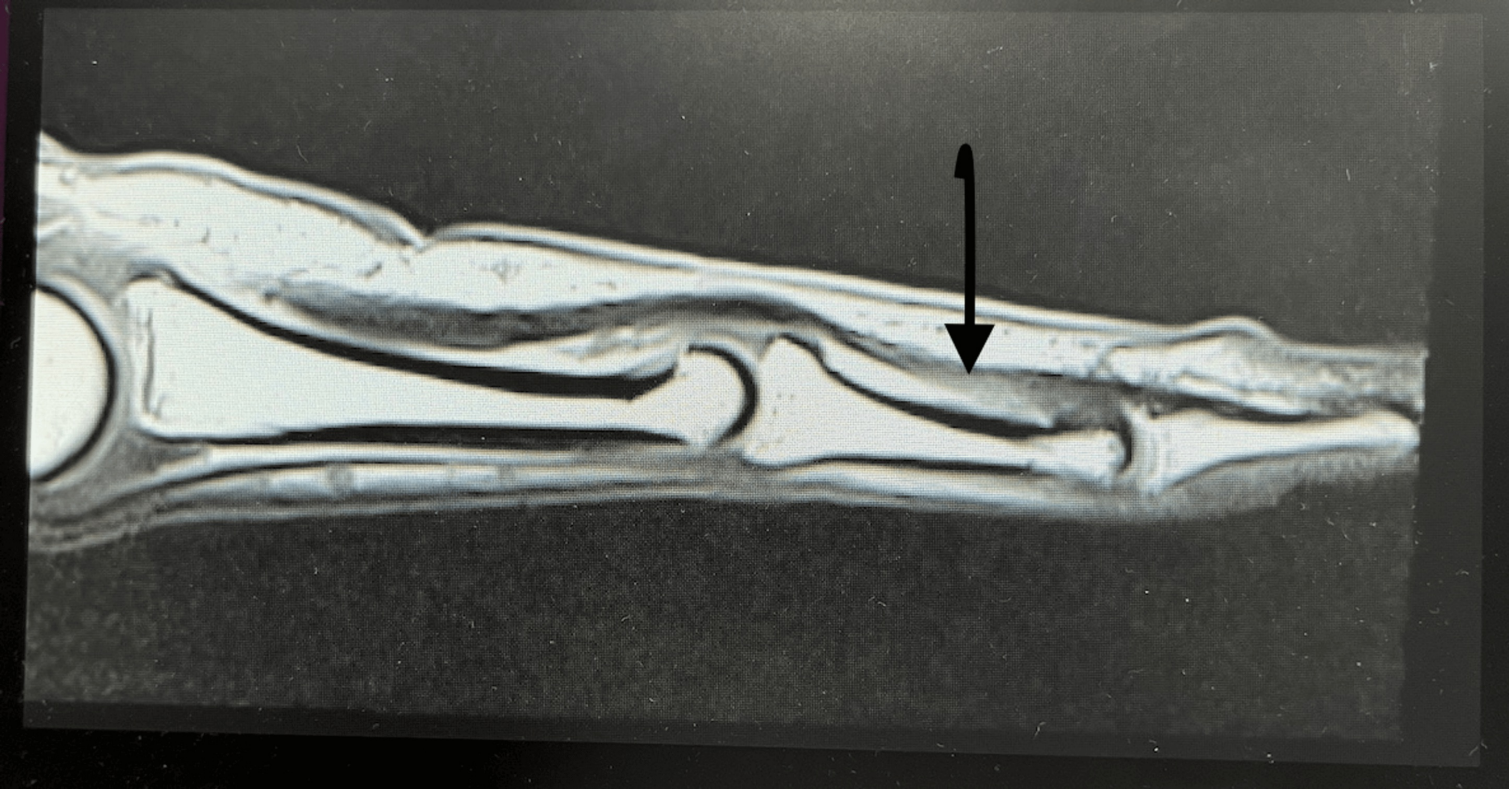
T1-weighted turbo spin echo sequence acquired in the sagittal plane of magnetic resonance imaging at WL 182 and slice thickness 2.5 mm, showing absence of FDPt in index finger Arrow shows the insertion of FDSt near the head of the middle phalanx, which is distal to its normal insertion. There is no FDPt. FDSt - flexor digitorum superficialis tendon; FDPt - flexor digitorum profundus tendon; WL - window level

**Figure 3:**
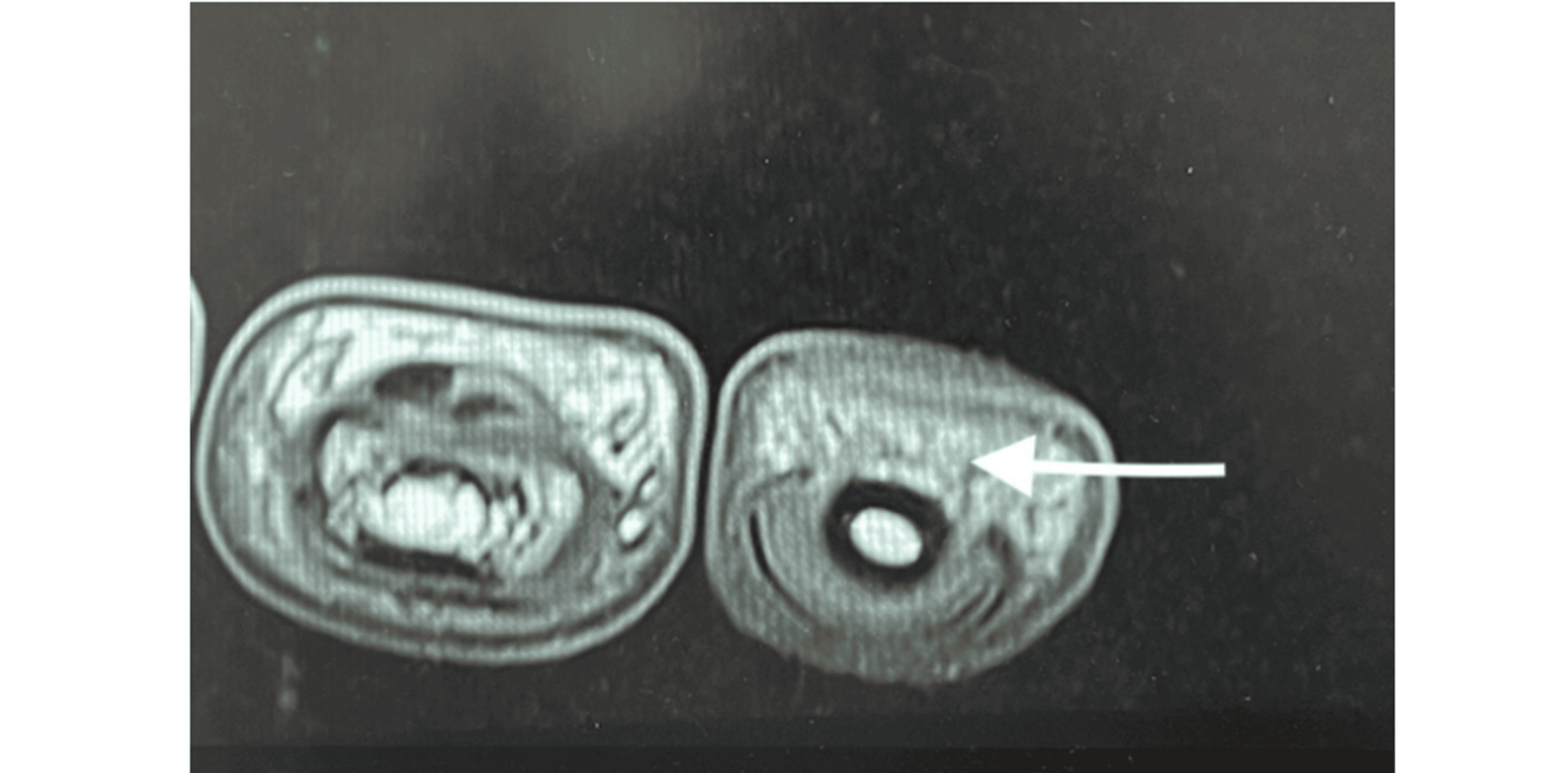
T1-weighted turbo spin echo sequence acquired in the transverse plane of magnetic resonance imaging at WL 413 and slice thickness 2.5 mm, showing absence of FDPt in index finger, at the level of distal phalanx. FDPt - flexor digitorum profundus tendon; WL - window level

The scald burns were managed conservatively at that time, without any surgical intervention or splinting. He also gave a history of inability to flex the right index finger distal interphalangeal joint since birth. There was no history of any other trauma, infection, or surgery. On examination, there was a flexion contracture of the right ring and little finger across the proximal interphalangeal joints. The girth of the distal phalange of the index finger was reduced compared to that of the opposite finger. There was an absence of the distal digital crease of the right index finger. Passive range of flexion and extension was normal, but there was no active flexion of the distal interphalangeal joint of the index finger. The function and strength of the FDSt, the lumbricals, and the extensor apparatus were normal. The range of motion of the distal interphalangeal joint was zero, and that of the proximal interphalangeal and metacarpophalangeal joints was normal. Grip strength was normal. Sensations and circulation were normal. On magnetic resonance imaging, the FDSt of the index finger was inserted a little distally, reaching up to the head of the middle phalanx (Figure 2, 3).

No discernible FDPt was present in the index finger. FDSt and FDPt in the rest of the fingers were normal.

Informed consent was taken for photography, publication of photographs the and surgery. The patient wanted correction of the flexion contractures of the ring and little fingers and refused any treatment for the index finger. The flexion contractures were released with multiple Z-plasties. Sutures were removed on day seven. Scar massage with emollient ointment was started on day 21. Postoperative recovery was uneventful, resulting in a full range of motion of the ring and little fingers and a more functional, cosmetically appealing hand. The patient was lost to follow-up after two months.

## Discussion

The absence of FDPt is a rare finding. Our search of PubMed and Google with terms 'absent flexor digitorum profundus', 'absent flexors hand', 'deficient flexor tendons', 'congenital lack of flexion', without any time filter, unearthed only eleven studies describing the FDPt absence. Eight of the eleven articles report an absent FDPt of the little finger in a total of nine patients/cadavers [1-9]. One case report describes the absence of FDPt of the middle finger, index, and ring finger, each [1, 10]. So our case, to our knowledge, is the second description of absent FDPt in the index finger.

Prevalence of absent FDSt is rather common (12-21%) [3]. Frohse, in 1908, was the first to document the absence of the FDSt and FDPt in a book titled 'Handbuch der Anatomie des Menschen' [7]. In the present case, the patient sought treatment for post-burn contractures of the ring and little fingers, and our watchful eyes detected the congenital tendon anomaly. In the cases reported by Kisner, the anomaly was detected during surgical exploration of accidental injury to the hand [5]. Four cases of FDPt absence were identified during cadaveric dissections [1,4,6]. This reinforces the notion that these anatomical variations are more prevalent than clinically appreciated [1].

One of the reasons for the inconspicuousness of this anomaly is the retained functionality of the hand [9]. Variant insertion of the FDSt to the distal phalanx could mask an absent FDPt by substituting its function at the distal interphalangeal joint. Hyatt et al. described a tendinous connection between FDSt and FDPt, which fully replaced the function of the missing FDPt [10]. A peculiar anatomy was noted by Kisner, where the sublimus tendon divided at the distal palmar crease to form a tendon akin to profundus [5]. Yilmaz et al. noted the anomalous tendon originating from the FDSt muscle in the forearm [6]. Besides anatomical substitutions, many patients develop functional substitutes by using the other structures to compensate for the lack of one. Our patient would use the adjoining middle finger FDPt to push the index finger distal interphalangeal joint into a flexed position, while gripping objects.

Absence of one tendon may not significantly impair hand function [3]. However, in cases where surgical intervention is planned, preoperative awareness of such anomalies is crucial to prevent intraoperative complications and litigation later. The concurrent absence of FDSt and FDPt can lead to notable grip weakness and limited digital flexion, necessitating targeted interventions [3]. Tendon transfer procedures, utilizing adjacent FDPt or tendon graft, may be required to restore flexion of the distal interphalangeal joint. Fukuoka et al imbricated the distal end of the FDPt stump to the neighbouring FDPt [2]. Two-stage palmaris longus grafting has been used to achieve a full range of motion of the index finger.

## Conclusions

To conclude, congenital absence of FDPt is an uncommon occurrence. Being asymptomatic, people usually present when there is a superadded injury or deformity, and not merely for the correction of this anomaly. The absence of the digital crease and reduced girth should raise suspicion, which should be followed by asking leading questions. Our case highlights the necessity of thorough clinical and radiological assessment in patients presenting with congenital variations.
